# A new mathematical model of bacterial interactions in two-species oral biofilms

**DOI:** 10.1371/journal.pone.0173153

**Published:** 2017-03-02

**Authors:** Bénédicte Martin, Zohreh Tamanai-Shacoori, Julie Bronsard, Franck Ginguené, Vincent Meuric, Fabrice Mahé, Martine Bonnaure-Mallet

**Affiliations:** 1 EA 1254 Microbiologie Risques infectieux, Université de Rennes 1, Université Européenne de Bretagne, Rennes, France; 2 Institut de Recherche Mathématique de Rennes, Université de Rennes I, CNRS, Université Européenne de Bretagne, Rennes, France; 3 Centre hospitalo-universitaire, Rennes, France; New York Medical College, UNITED STATES

## Abstract

Periodontitis are bacterial inflammatory diseases, where the bacterial biofilms present on the tooth-supporting tissues switch from a healthy state towards a pathogenic state. Among bacterial species involved in the disease, *Porphyromonas gingivalis* has been shown to induce dysbiosis, and to induce virulence of otherwise healthy bacteria like *Streptococcus gordonii*. During biofilm development, primary colonizers such as *S. gordonii* first attach to the surface and allow the subsequent adhesion of periodontal pathogens such as *P. gingivalis*. Interactions between those two bacteria have been extensively studied during the adhesion step of the biofilm. The aim of the study was to understand interactions of both species during the growing phase of the biofilm, for which little knowledge is available, using a mathematical model. This two-species biofilm model was based on a substrate-dependent growth, implemented with damage parameters, and validated thanks to data obtained on experimental biofilms. Three different hypothesis of interactions were proposed and assayed using this model: independence, competition between both bacteria species, or induction of toxicity by one species for the other species. Adequacy between experimental and simulated biofilms were found with the last hypothetic mathematical model. This new mathematical model of two species bacteria biofilms, dependent on different substrates for growing, can be applied to any bacteria species, environmental conditions, or steps of biofilm development. It will be of great interest for exploring bacterial interactions in biofilm conditions.

## Introduction

Biofilms are complex and organized bacterial communities attached to a substratum and embedded in an adhesive and protective matrix. In nature, mixed biofilms, i.e. composed of different species are predominant. In biofilms, interactions between bacteria are essential, either additive, synergistic or competitive or even detrimental interactions. Bacteria can interact via physical interactions by specific adhesive proteins, by signaling pathways, or by metabolic interactions [1]. In the latter, bacteria can exchange nutrients necessary for their survival or growth, but also process nutrients in such a way that other bacteria species can use them [2]. In detrimental interactions, bacterial products can be deleterious or even toxic for other species.

Periodontitis is an inflammatory disease initiated by oral microbial biofilm, relying on a physiological shift of the oral microbiome toward a virulent state, in response to host environment [3]. Periodontitis biofilms are composed of various bacteria species. Early colonizers of the salivary pellicle on the tooth surfaces, mainly commensal oral streptococci such as *Streptococcus gordonii*, initiate biofilm formation by favoring adhesion and colonization by late pathogenic colonizers, including *Porphyromonas gingivalis*. *P. gingivalis* a Gram-negative, black-pigmented, anaerobic rod, is now widely accepted as a keystone pathogen [4–6]: despite its low-abundance, *P. gingivalis* can lead the process of periodontal inflammation and tissue destruction by transforming a normally healthy microbiome into a dysbiotic state. Recent metagenomic and metatranscriptomic data obtained from healthy or periodontitis-associated tissues confirmed the involvement of *P. gingivalis* in periodontal disease progression [7, 8].

In the mouth, either in healthy or periodontitis-associated tissues, *P. gingivalis* is always encountered in streptococcal sites [9]. *P. gingivalis* is able to adhere and colonize specifically *S. gordonii* biofilms [10]. Whereas Streptococcus species are usually linked to healthy status of the gingiva [11], recent data suggested that commensal species can play a role in expression of virulence in periodontal diseases. Expression profiles of these species can be modified in progressing periodontal tissues as compared with non-progressing tissues [7]. Moreover, studies performed in in vitro biofilm models also showed that addition of periodontopathogens such as *P. gingivalis* to biofilms composed of commensal species induced a shift in the expression profile in streptococcal species [12]. Finally, experiments performed in animal mice models proved that virulence of *P. gingivalis* was increased by *S. gordonii*, as evidenced by the enhanced alveolar bone loss observed after inoculation of both bacteria as compared to *P. gingivalis* alone [13]. Interactions between both species have been described, either metabolic or signaling interactions [14]. Whereas *S. gordonii* metabolism is based on the fermentation of carbohydrates, *P. gingivalis* relies on oligo-peptides [15, 16] and/or amino acid such as Arginine [17] to produce energy. To obtain peptides and amino-acids, *P. gingivalis* bacteria produce specific proteases that can degrade [18] or cleave glycoproteins [19].

Published studies on mixed *P. gingivalis*-*S. gordonii* biofilms focused on the first steps of biofilm development, and especially *P. gingivalis* recruitment by *S. gordonii* [20]. Genes responsible for co- adhesion between both species and essential for mixed biofilm formation have been identified, and are mainly involved in inter-species signaling [21–23]. Modifications of bacterial metabolism in each species were also pointed out by proteomic studies performed in early steps of two-species biofilm development [24, 25].

However, little information is available regarding the next steps of biofilm development, namely growth and maturation. The present work was therefore focused on the first growing step of two-species *P. gingivalis* and *S. gordonii*-biofilms development, when mortality was still negligible. The objective was to understand the influence of interactions between both bacterial species on the growth of the biofilm, thanks to a mathematical model established from experimental data.

Different approaches have been proposed to model biofilm growth. Published methods described the species composition of the biofilm without spatial data [26], while others explored variations in the depth of the structure [27]. Recently a 1D mathematical model of the dental plaque has been proposed [28]. Two dimensional or even 3D methods give access to the spatial structure of the biofilm, such as continuous mechanical models [29] or discrete individual-based models [30] and cellular automata [31].

In this work, a 2D individual based Cellular automata, describing width and height of the biofilm, was chosen to model the growth of two species-biofilm. Each bacterium was considered as an individual characterized by a particular status and behavior. This kind of model allows a good description of the biofilm with a reasonable computing cost. Biofilm growth is highly dependent on substrate availability for bacteria. In the mouth, different micro-environments can provide bacteria with various nutrient concentrations, oxygen availability and pH levels. Substrate concentrations in different parts of the biofilm rely on substrate initial concentration and on substrate biochemical properties which influence substrate diffusion in biofilms. Growth of bacteria in biofilms also depends on bacteria growth characteristics and energy metabolism. These different parameters, inherent either to substrate or to bacteria species were integrated in the mathematical model described in this work. An additional parameter was introduced in the biofilm model, defined as a damage parameter which can lead to limit cell growth and even lead to cell dispersal, without decreasing viability. Production and decay of damage within bacteria were shown to affect biofilm structure and development in [32]. Calibration of growth, substrate, and damage parameters was allowed by experimental measurements or estimation by inverse problem in mono-species biofilms. Thanks to the model developed, the nature of the interactions between both species in biofilm was studied and three different hypothesis were tested: (1) independence or (2) competition for substrate between both species and (3) production of toxic molecules by one species. The mathematical model was validated by comparing simulated and experimental results for two species biofilms.

## 1 Methods

### 1.1 Mathematical model

#### 1.1.1 Simulation domain and cellular automata

**Fig 1:**
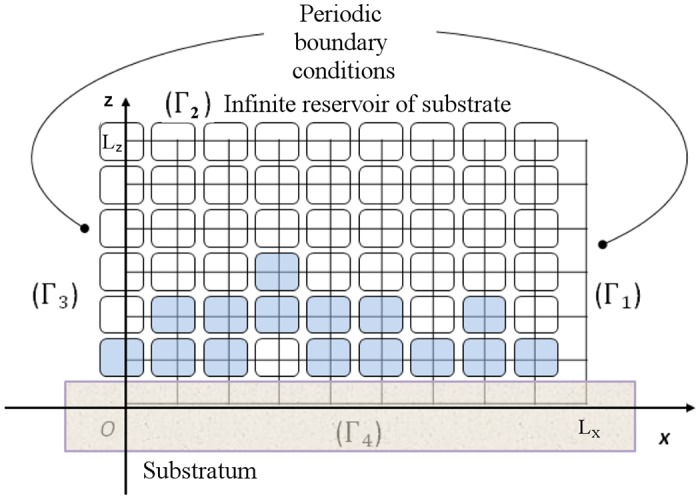
Computing domain [0, *L*_*x*_] × [0, *L*_*z*_] and grid of the cellular automata.

The development of the biofilm is modeled by a cellular automata [31, 33] using a 2D-lattice, representing width and height of the biofilm, to discretize the environment (see Fig 1). Each element in the lattice can contain one bacterial cell. The bacterial biomass in this element is given by the biomass density $c_{x}^{\left(i\right)}$ at the center of the element. The concentration of the substrate is computed at the same points of the domain. When the biomass density becomes greater than the maximum biomass concentration $c_{xm}^{\left(i\right)}$, the bacterium divide into two cells. One cell remains in the same place and the other is placed randomly in a free neighboring element. If there is no free neighboring element, the cell randomly replaces one of the neighboring cells and the process is repeated with the replaced cell until a free element is found.

The biofilm grows on a planar substratum. The reservoir of substrate above the substratum is supposed to be large as compared to the height of the biofilm, so that the reservoir of substrate above the biofilm is considered infinite. The height *L*_*z*_ of the computing domain *Ω* = [0, *L*_*x*_] × [0, *L*_*z*_] is set to be equal to sum of the height of the biofilm and the thickness *l*_*b*_ = 18 *μm* of a diffusive boundary layer. The boundary conditions for computing the concentration of the substrate $c_{s}^{\left(i\right)}\left(t,x,y\right)$ are the following (see Fig 1): the concentration of the substrate $c_{s}^{\left(i\right)}$ on the top of the domain Γ_2_ is equal to its constant value $c_{s0}^{\left(i\right)}$ in the infinite reservoir, the effect of the substratum is modeled by a zero-flux boundary condition, $\frac{\partial c_{s}^{\left(i\right)}}{\partial z}=0$, on Γ_4_, and a periodic condition is used on the lateral boundaries Γ_1_ and Γ_3_ to avoid edge effects.

According to experimental dimensions, the width of the domain *L*_*x*_ is set at 123 *μm*. To contain one bacterial cell and its extracellular constituents the size of the volume element of the lattice Δ*x* is set at 1.23 *μm* and there are 100 elements in each row of the two-dimensional grid.

#### 1.1.2 Definition of substrate, bacteria and damage parameters

$c_{s}^{\left(i\right)}\left(t,x,z\right)$ is defined as the concentration of the substrate for the bacterium *i*, *i* = 1 for *P. gingivalis* and *i* = 2 for *S. gordonii*, at the time *t* and the spatial point of coordinates (*x*, *z*).

A set of specific parameters is associated to each bacterial cell: the maximum specific growth rate $\mu_{m}^{\left(i\right)}$, the yield coefficient $Y_{xs}^{\left(i\right)}$ which measures the efficiency of the transformation of the substrate in bacterium biomass, the maintenance coefficient $m_{s}^{\left(i\right)}$, the maximum biomass concentration $c_{xm}^{\left(i\right)}$. Mean values of these coefficients are reported in Table 1. When a new bacterium is created, a set of values is generated by random draws with a Gaussian distribution around the mean values given in the Table 1 and with a standard deviation of 5% [34]. At the beginning of the simulation, to model the adhesion of bacteria on the substratum, $N_{0}^{\left(i\right)}$ bacteria are randomly placed on the substratum without any damage and with a biomass given by an uniform random draw between $\frac{c_{xm}^{\left(i\right)}}{2}$ and $c_{xm}^{\left(i\right)}$.

**Table 1:** Model parameters and variables.

Symbol	Description	Values	Unit
Δ*t*	Time step	3600	s
Δ*x*	Spatial element size	1.23 ⋅ 10^−6^	m
*L*_*x*_	Width of the domain	123 ⋅ 10^−6^	m
*l*_*b*_	Thickness of the diffusive boundary layer	18 ⋅ 10^−6^	m
*P. gingivalis*: *i* = 1			
$c_{s}^{\left(1\right)}$	Substrate concentration (proteins)		*kg*_*s*_ *m*^−3^
$c_{s0}^{\left(1\right)}$	Substrate concentration in the reservoir	16	*kg*_*s*_ *m*^−3^
$K_{s}^{\left(1\right)}$	Half saturation coefficient	6.1609	*kg*_*s*_ *m*^−3^
$D_{s}^{\left(1\right)}$	Diffusion coefficient	5 ⋅ 10^−11^	*m*^2^ *s*^−1^
$c_{x}^{\left(1\right)}$	Biomass concentration		*kg*_*x*_ *m*^−3^
$c_{xm}^{\left(1\right)}$	Maximum biomass concentration	38	*kg*_*x*_ *m*^−3^
$\mu_{m}^{\left(1\right)}$	Maximum specific growth rate	6.62 ⋅ 10^−5^	*s*^−1^
$Y_{xs}^{\left(1\right)}$	Growth yield coefficient	0.18	$kg_{x}\;kg_{s}^{-1}$
$m_{s}^{\left(1\right)}$	Maintenance coefficient	4.49 ⋅ 10^−5^	$kg_{s}\;kg_{x}^{-1}\;s^{-1}$
$N_{0}^{\left(1\right)}$	Initial number of elements containing biomass	175	
*S. gordonii*: *i* = 2			
$c_{s}^{\left(2\right)}$	Substrate concentration (glucose)		*kg*_*s*_ *m*^−3^
$c_{s0}^{\left(2\right)}$	Substrate concentration in the reservoir	2	*kg*_*s*_ *m*^−3^
$K_{s}^{\left(2\right)}$	Half saturation coefficient	0.1091	*kg*_*s*_ *m*^−3^
$D_{s}^{\left(2\right)}$	Diffusion coefficient	57 ⋅ 10^−11^	*m*^2^ *s*^−1^
$c_{x}^{\left(2\right)}$	Biomass concentration		*kg*_*x*_ *m*^−3^
$c_{xm}^{\left(2\right)}$	Maximum biomass concentration	44	*kg*_*x*_ *m*^−3^
$\mu_{m}^{\left(2\right)}$	Maximum specific growth rate	2.6733 ⋅ 10^−4^	*s*^−1^
$Y_{xs}^{\left(2\right)}$	Growth yield coefficient	0.038	$kg_{x}\;kg_{s}^{-1}$
$m_{s}^{\left(2\right)}$	Maintenance coefficient	5 ⋅ 10^−4^	$kg_{s}\;kg_{x}^{-1}\;s^{-1}$
$N_{0}^{\left(2\right)}$	Initial number of elements containing biomass	315	

All parameters were measured experimentally or derived form experimental results by inverse problem, except for the lattice parameters (Δ*x*, *L*_*x*_, *l*_*b*_) which were arbitrarily set up to values compatible with microscopy studies.

*w*^(*i*)^ is the damage concentration in the bacterium *i*.

#### 1.1.3 Model of substrate diffusion and reaction in mono-species biofilms

The biomass density of the bacterium *i* being depicted by the variable $c_{x}^{\left(i\right)}$, the dynamics of concentration of the substrate $c_{s}^{\left(i\right)}$ is given by the following reaction-diffusion equation

$$\begin{array}{c} \frac{\partial c_{s}^{\left(i\right)}}{\partial t}=D_{s}^{\left(i\right)}▵c_{s}^{\left(i\right)}-r_{s}^{\left(i\right)}\left(c_{s}^{\left(i\right)},c_{x}^{\left(i\right)}\right) \end{array}$$ (1)

where $▵=\frac{\partial^{2}}{\partial x^{2}}+\frac{\partial^{2}}{\partial z^{2}}$ is the Laplace operator, $D_{s}^{\left(i\right)}$ is the diffusion coefficient and $r_{s}^{\left(i\right)}\left(c_{s}^{\left(j\right)},c_{x}^{\left(i\right)}\right)$ represents the rate of substrate consumption by the bacterium. This consumption rate is depending on the biomass concentration and the substrate concentration at the considered point as follows

$$\begin{array}{c} r_{s}^{\left(i\right)}\left(c_{s}^{\left(i\right)},c_{x}^{\left(i\right)}\right)=(\frac{\mu_{m}^{\left(i\right)}}{Y_{xs}^{\left(i\right)}}+m_{s}^{\left(i\right)})c_{x}^{\left(i\right)}\frac{c_{s}^{\left(i\right)}}{K_{s}^{\left(i\right)}+c_{s}^{\left(i\right)}} \end{array}$$ (2)

where $K_{s}^{\left(i\right)}$ is the half-saturation coefficient.

Variations of the damage concentrations are governed by the following equation

$$\begin{array}{c} \frac{\partial w^{\left(i\right)}}{\partial t}=\alpha^{\left(i\right)}[r_{s}^{\left(i\right)}\left(c_{s}^{\left(i\right)},c_{x}^{\left(i\right)}\right)+m_{s}^{\left(i\right)}c_{x}^{\left(i\right)}]-\beta^{\left(i\right)}w^{\left(i\right)}, \end{array}$$ (3)

where *α*^(*i*)^ is the damage conversion factor and *β*^(*i*)^ is the rate of damage removal. The damages are not measured experimentally but are used in the model as a theoretical tool to take into account different causes of the alteration of growth process.

Substrate consumption by bacteria allow bacteria not only to grow but also to sustain for their endogenous metabolism $m_{s}^{\left(i\right)}c_{x}^{\left(i\right)}$. The efficiency of the transformation of the substrate in bacterium biomass is supposed to decreases when the level of damage increases in the bacterium. The following equation allows to describe dependence between those different biological processes

$$\begin{array}{c} \frac{\partial c_{x}^{\left(i\right)}}{\partial t}=\frac{Y_{xs}^{\left(i\right)}}{1+e^{\gamma^{\left(i\right)}\left(w^{\left(i\right)}-\delta^{\left(i\right)}\right)}}(r_{s}^{\left(i\right)}\left(c_{s}^{\left(i\right)},c_{x}^{\left(i\right)}\right)-m_{s}^{\left(i\right)}c_{x}^{\left(i\right)}) \end{array}$$ (4)

where the intensity of the effect of the damages on the growth of the bacterium is governed by the parameters *γ*^(*i*)^ and *δ*^(*i*)^. Values of these parameters are given in Table 2.

**Table 2:** Damage parameters and variables.

Symbol	Description	Values	Unit
*P. gingivalis*: *i* = 1			
*w*^(1)^	Damage concentration		*kg*_*w*_ *m*^−3^
*α*^(1)^	Damage conversion factor	2.78 ⋅ 10^−5^	$kg_{w}\;kg_{s}^{-1}$
*β*^(1)^	Rate of damage removal	1.2 ⋅ 10^−6^	*s*^−1^
*γ*^(1)^	Coefficient 1 of damage effect	300	$kg_{w}^{-1}\;m^{3}$
*δ*^(1)^	Coefficient 2 of damage effect	9.01 ⋅ 10^−2^	*kg*_*w*_ *m*^−3^
*S. gordonii*: *i* = 2			
*w*^(2)^	Damage concentration		*kg*_*w*_ *m*^−3^
*α*^(2)^	Damage conversion factor	5 ⋅ 10^−5^	$kg_{w}\;kg_{s}^{-1}$
*β*^(2)^	Rate of damage removal	3.45 ⋅ 10^−5^	*s*^−1^
*γ*^(2)^	Coefficient 1 of damage effect	300	$kg_{w}^{-1}\;m^{3}$
*δ*^(2)^	Coefficient 2 of damage effect	6.916 ⋅ 10^−2^	*kg*_*w*_ *m*^−3^
Toxic substance for *P. gingivalis* produced by *S. gordonii*			
*v*^(2)^	Toxic substance concentration		*kg*_*v*_ *m*^−3^
*η*^(2)^	Toxic substance production factor	2.5 ⋅ 10^−14^	$kg_{v}\;kg_{x}^{-1}\;s^{-1}$
*ζ*^(2)^	Rate of damage production	1	$kg_{w}\;kg_{v}^{-1}\;s^{-1}$

The Eqs (3) and (4) are solved by an explicit Euler method with a time step Δ*t* = 1 *h*. Since the diffusion of the substrate is much faster than the growth of the biomass, we solve the stationary equation corresponding to Eq (1) after each modification of the biomass. We use the classical second order central finite difference method with the step Δ*x* to solve it. At the beginning, the substrate concentration is initialized to $c_{s0}^{\left(i\right)}$ in the whole domain.

The Models (1)–(4) allows to study the growth of mono-bacterial biofilm.

#### 1.1.4 Simulation of interaction between bacteria species in two-species biofilms

To study the interaction of the two species in a same biofilm, 3 different hypothesis were tested by different models: independence, competition for nutrients and production of toxic molecules by one species.

In the first hypothesis (independence), the limiting nutrient is not the same for each bacterium: proteins for *P. gingivalis* and glucose for *S. gordonii*. In this case, there is no competition for the nutrients between bacteria species. This hypothesis can be tested with Eqs (1)–(4) solved together for the both species.

In the second hypothesis (competition for nutrients), the limiting nutrient is supposed to be the same for each bacterium. The mathematical model is still defined by the Eqs (1)–(4) for *i* = 1 and *i* = 2 but with $c_{s}^{\left(1\right)}=c_{s}^{\left(2\right)}$.

In the last hypothesis, the model is implemented with the diffusion of a substance *v*^(2)^ produced by one species (*S. gordonii*) and which contributes to increase the damage in the other species (*P. gingivalis*). The following equation describes the kinetics of the concentration of the toxic substance *v*^(2)^

$$\begin{array}{c} \frac{\partial v^{\left(2\right)}}{\partial t}=D_{v}^{\left(2\right)}▵v^{\left(2\right)}+\eta^{\left(2\right)}c_{x}^{\left(2\right)}. \end{array}$$ (5)

The Eq (3) was also modified to account for the *v*^(2)^-dependent damage production in *P. gingivalis*:

$$\begin{array}{c} \frac{\partial w^{\left(1\right)}}{\partial t}=\alpha^{\left(1\right)}[r_{s}^{\left(1\right)}\left(c_{s}^{\left(1\right)},c_{x}^{\left(1\right)}\right)+m_{s}^{\left(1\right)}c_{x}^{\left(1\right)}]-\beta^{\left(1\right)}w^{\left(1\right)}+\zeta^{\left(2\right)}v^{\left(2\right)} \end{array}$$ (6)

where *η*^(2)^ is the toxic substance production factor and *ζ*^(2)^ the rate of damage production.

### 1.2 Experimental biofilms

#### 1.2.1 Bacterial strains

*P. gingivalis* ATCC 33277 and *S. gordonii* DL1, were grown on blood Columbia agar plates and/or in a brain-heart infusion broth (BHIe) (Biomérieux, France) supplemented with menadione (10 *μ*g.mL^−1^) and hemin (5 *μ*g.mL^−1^) (Sigma, Saint Quentin Fallavier, France). Protein concentrations were measured by BCA colorimetric assay (Pierce Protein assay kit, Rockford, USA) to deduce protein consumption by bacteria. In BHIe, glucose concentration was 2g.L-1. For each experiment, cultures were used in the middle of log-phase growth at 37°C in an anaerobic chamber (MAC 500, Don Whitley Scientific, Shipley, UK) with 10% H_2_, 10% CO_2_, and 80% N_2_.

#### 1.2.2 Determination of bacterial growth parameters

*S. gordonii* and *P. gingivalis* cultures were inoculated from fresh colonies and incubated overnight at 37°C in BHIe. Cultures were then diluted in 10 mL of the same medium at optical density at 600 nm (OD_600_) of 0.1 ± 0.05 and incubated in an anaerobic chamber at 37°C. OD600 were read every hour for 12 h and then, every 6 h until stationary/death phase was reached. Experiments were performed either in pure or diluted BHIe. Growth curves were established from OD readings from triplicate biological samples and allowed to measure growth parameters such as the specific growth rate *μ*. The half-saturation coefficient KS and the maximal specific growth rate *μ*_*m*_ were estimated from the *μ* calculated at various dilutions of BHIe and the corresponding substrate concentration in BHIe.

#### 1.2.3 Biofilm formation

Exponential bacterial cultures of *S. gordonii*, *P. gingivalis* were harvested. Biofilm were grown in sterile Ludin^®^ chambers (Life Imaging Services, Switzerland) connected to a peristaltic pump (Minipuls 3, Gilson, Middleton, WI) allowing a flow rate of 7 mL.h^−1^ through silicone tubing in anaerobic conditions. Flow cells were coated with 0.22-*μ*m filtrated sterile human saliva (collected from at least six healthy volunteers, treated with 2.5mM dithiothreitol and diluted in distilled water to obtain a 25% (v/v) solution) for 30 min before bacteria inoculation. All steps (inoculation, washing, staining) were performed with a flow rate of 7mL.min^−1^.

For assays of mono or dual-species biofilm formation, *P. gingivalis* (OD_600_ = 0.1, ie 2 ⋅ 10^8^ CFU/mL), *S. gordonii* (OD_600_ = 0.02, ie 2 ⋅ 10^7^ CFU/mL) or both species (*P. gingivalis* OD_600_ = 0.1, *S. gordonii* OD_600_ = 0.02) were inoculated for 15 minutes in the flowing system and left in anaerobic conditions at 37°C without flow until use for characterization. At each time point (3, 24, 48, 72 or 120 hours), biofilms were used once for studies by confocal scanning microscopy and/or by fluorescence correlation spectroscopy. For each time point, experiments were repeated at least three times.

#### 1.2.4 Biofilm characterization by confocal scanning microscopy

After washing with PBS for 15 minutes, biofilms were stained with 5 *μ*M of Syto^®^40 nucleic acid dye (Molecular Probes, Lieden, The Netherlands) diluted in PBS for 15 minutes. Flow cells were then observed *in situ* with a Leica TCS-SP8 confocal laser scanning microscope (Leica Microsystems, Wezlar, Germany). An HC PL Apo 63X, 1.4 NA, oil immersion objective lens was used for image capture and a numerical zoom of 1.5 was applied. The 405-nm UV diode and a 420 to 500-nm band-pass emission filter were used to detect all bacteria stained with Syto^®^40.

Biofilm stacks (123 × 123 *μ*m) acquired at 0.5 *μ*m intervals were scanned with a line average of 2. Leica software (LAS AF V.2.2.1) was used for microscope piloting and image acquisition. Analysis of images based on Syto^®^40 fluorescence intensity levels were performed in Comstat 2 plugin in ImageJ software V1.43m (National Institute of Health) to estimate characteristic parameters of biofilms: the biomass, representing the overall volume of the biofilm (expressed in *μm*^3^/*μm*^2^), the average thickness (defined as the average of thicknesses over given locations, ignoring pores and voids inside the biofilm), the roughness coefficient (calculated from the thickness distribution) which is an indicator of biofilm heterogeneity, and the surface to volume ratio (the surface area divided by the biomass) which reflects what fraction of the biofilm is exposed to the nutrient flow. All these parameters are described in [35].

#### 1.2.5 Diffusion measurements in biofilms by fluorescence correlation spectroscopy

Biofilms composed of *P. gingivalis* alone, *S. gordonii* alone or both species together were used in these experiments with two diffferent fluorescent molecules used to study diffusion by FCS. Biofilms were washed for 3 hours with Phosphate Buffer Saline (PBS) before incubation with either 10nM SNAP-Cell^®^TMR-Star or 1nM AlexaFluor555-Goat anti Rabbit IgG antibody (Molecular Probes, Lieden, The Netherlands) for 20 minutes. Control experiments showed that, in the experimental conditions used (time and concentration exposure of molecules), none of the fluorescent molecules was detected inside the bacterial cells and was interfering with the FCS analysis. Biofilms were then analyzed by FCS using a Leica TCS-SP8 confocal laser scanning microscope (Leica Microsystems, Wezlar, Germany) equipped with a HC PL Apo 63X, 1.2 NA, water immersion objective lens with a numerical zoom of 6. For both TMR-Star and AlexaFluor555-conjugated antibody, excitation was performed using a 561-nm diode laser and fluorescence intensity fluctuations were recorded by avalanche photodiode detectors (Tau-SPAD, PicoQuant) in the emission ranges of 581-684nm. Leica software (LAS AF V.2.2.1) was used for microscope piloting and acquisition of fluorescence intensity fluctuations were recorded for 30 seconds using SymphoTime software (PicoQuant). Diffusion was assessed at 5 *μ*m deepness from the surface in at least 8 random positions in the *xy* plane of biofilms. Analysis of autocorrelation curves was performed using SymphoTime software, assuming a 3D Gaussian distribution of the fluorophores in the confocal volume. For diffusion coefficient determination ($D_{s}^{\left(i\right)}$), confocal volume and excentricity parameters were first estimated from point spread function measurements specific to the confocal microscope. Curve fitting by SymphoTime software was performed according to triplet state model, with confocal volume and excentricity parameters fixed. Evaluation of triplet state time was achieved in PBS and used for all subsequent fitting in biofilm samples. Curve fitting applied with a Triplet State Model allowed calculation of diffusion time, diffusion coefficient and anomality coefficient at each sample biofilm position.

#### 1.2.6 Quantification of bacteria by RT-PCRq

DNA was extracted from biofilms using the QIAamp DNA Mini kit (Qiagen) according to manufacturer’s protocol with minor modifications: after centrifugation at 10 000g for 10 minutes, lysis step at 56°C using proteinase K was extended to 18 hours. Standard curves were established using DNA extractions of each bacterial species cultures (*P. gingivalis* and *S. gordonii*). Following extraction, DNA of all samples (standard and biofilms) was determined using a NanoDrop ND-1000 (ThermoFischer Scientific). DNA standards were set to defined concentrations ranging from 10 to 0.0001 ng per sample using 1:10 serial dilutions. The qPCR was run in a total reaction volume of 25 *μ*l containing 12.5 *μ*l Sybr Green Master Mix (Eurogentec), 1 *μ*l of each primer (5 *μ*M), and 2 *μ*l of sample. Amplification of the extracted DNA template was performed in an ABI 7000 Sequence Detection System (Applied Biosystems) by initial incubation of 2 min at 55°C and 10 min at 95°C, followed by 40 cycles of 15 sec at 95°C and 1 min at 60°C. A dissociation stage was added at the end of the qPCR run consisting of 15 sec at 95°C followed by 20 sec at 60°C and another 15 sec at 95°C. From the obtained threshold cycles (Ct), sample DNA concentrations were calculated for each organism using standard curves. Primers used for quantification targeted 16S ribosomal RNA gene and were specific of each species: forward and reverse primers sequences were respectively AAGCAACGCGAAGAACCTTA and TCCGAACTGAGACTGGCTTT for *S. gordonii*, and TGGGTTTAAAGGGTGCGTAG and CAATCGGAGTTCCTCGTGAT for *P. gingivalis*.

#### 1.2.7 Determination of cell size parameters by scanning electronic microscopy

Monobacterial suspensions of *P. gingivalis* or *S. gordonii* grown in BHIe medium were collected, washed 3 times with PBS and fixed with 2.5% glutaraldehyde/sodium cacodylate 0.1M, dehydrated by ethanol up to 100%, critical point dried and metallized with palladium-gold. Micrographs were acquired on a Scanning Electronic Microscope JSM 6301F (JEOL, Tokyo, Japan) at the Centre de Microscopie Électronique à Balayage et MicroAnalyse (CMEBA) of Rennes and length parameters of bacteria, such as cell diameters before or after division, were measured on at least 20 bacteria. Diameters were used to calculate maximal and mean bacterial volume for both species, which allow the estimation of the yield coefficient *Y*_*xs*_ and the maintenance coefficient *m*_*s*_ for each bacterial species with one specific susbtrate.

## 2 Results

### 2.1 Biological characterization of biofilms

#### 2.1.1 Biofilm architecture

**Fig 2:**
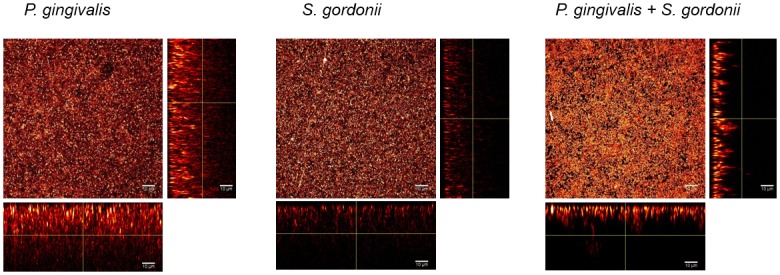
Typical fluorescence images of biofilms after 48 hours. *P. gingivalis*, *S. gordonii* or both species were inoculated for 15 min in a saliva-coated flow cell. After 48 hours, bacteria were stained with 5 *μ*M of Syto40 and flow cells were observed by confocal laser scanning microscopy. Images of optical stacks were processed using IMAGEJ software. Images of one representative experiment for each type of biofilm are shown, with the maximum 3D z projection of acquired stacks and the 2-D x-z or y-z planes slices.

Biofilms containing one or two species were followed from 3 to 120 hours by confocal microscopy. Typical fluorescence images are shown in Fig 2.

Biomass and average thicknesses of biofilms calculated from fluorescence intensities increased from 3 to 48 hours in all types of biofilms, before a decrease at 72 hours. However, these data reflected mainly living bacteria, as the extent of Syto40 staining is much lower in dead cells than in living cells.

**Fig 3:**
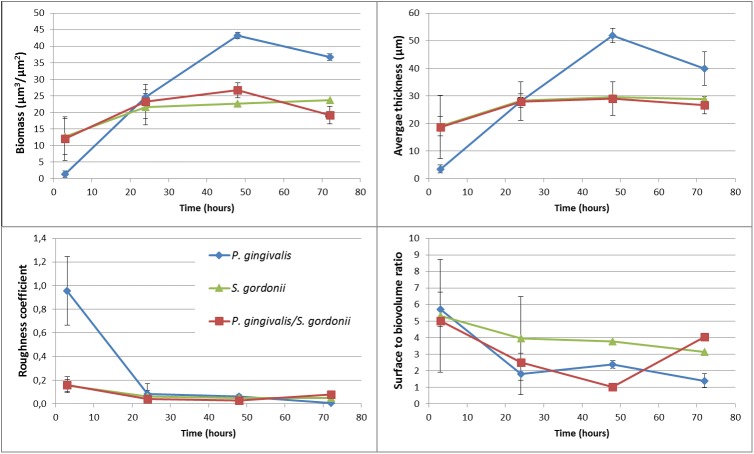
Kinetic evolution of biofilms specific parameters in biofilms. *P. gingivalis*, *S. gordonii* or both species were inoculated for 15 min in a saliva-coated flow cell. After 3, 24, 48, or 72 hours, bacteria were stained with 5 *μ*M of Syto40 and flow cells were observed by confocal laser scanning microscopy. Intensities from biofilm images were processed using IMAGEJ software implemented with Comstat2 plugin to calculate biomass, roughness coefficient, average thickness and surface to biovolume ratio parameters.

In mono-species *S. gordonii* biofilms, bacteria were homogeneously and densely distributed on the surface, so that roughness coefficients were low (Figs 2 and 3). The architecture of two-species *S. gordonii*-*P. gingivalis* biofilms was similar to mono-species *S. gordonii* biofilms. Indeed, estimation of bacteria numbers by PCRq after 48 hours (which quantified live and dead bacteria) showed that, even if both mono-species biofilms contained similar quantities of bacteria, *S. gordonii* were predominant in two species *P. gingivalis*/*S. gordonii* biofilms, with more than 99% identified as *S. gordonii*: *P. gingivalis* amounts of bacteria decreased from 1.18 ⋅ 10^9^ ± 1.27 ⋅ 10^9^ in monospecies *P. gingivalis* biofilms to 3.21 ⋅ 10^6^ ± 1.27 10^6^ in two species biofilms. *S. gordonii* amounts remained stable, with 1.08 ⋅ 10^9^ ± 8.01 ⋅ 10^8^ in both types of biofilms.

In contrast, in mono-species *P. gingivalis* biofilms, bacteria were more heterogeneously distributed throughout the surface and along the thickness of the biofilm (Fig 2). Roughness coefficients of these biofilms were higher than those found in mono-species *S. gordonii* biofilms or *S. gordonii*-*P. gingivalis* biofilms (Fig 3C), even if they tend to decrease with time.

As shown in Fig 3, the growth of *S. gordonii* mono-bacterial biofilms was fast but reached a plateau as soon as 24 hours with a maximal biofilm thicknesses of 30 *μ*m. In contrast, the growth of *P. gingivalis* mono-bacterial biofilms was slower but was still in an increasing phase after 48 hours. These biofilms were thicker than *S. gordonii* biofilms with up to 50 *μ*m-thicknesses obtained after 48 hours.

The surface to bio-volume ratio, reflecting the fraction of biofilm exposed to nutrient solution, decreased in the first hours after adhesion of bacterial cells until 24 hours and remained stable until at least 72 hours in the homotypic *P. gingivalis* biofilms (Fig 3D). In contrast, in both *S. gordonii*-containing biofilms, these ratios decreased from 2 to 48 hours and increased at 72 hours.

#### 2.1.2 Diffusion in biofilms

To evaluate substrate availability in biofilms, diffusion characteristics of two different fluorescent molecules were studied, varying in size and fluorochrome type: an AlexaFluor555-conjugated IgG anti-rabbit antibody was used to mimic protein behavior and a smaller molecule, TMR-Star was used to simulate diffusion of small peptides or sugars.

**Fig 4:**
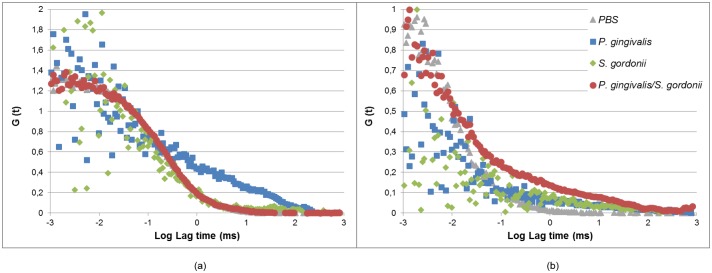
Effect of bacterial composition on diffusion properties in biofilms. Typical autocorrelation curves obtained at 5 *μ*m deepness in 48 hours-biofilms with (a) 1nM AlexaFluor555-conjugated IgG anti-rabbit antibody or (b) 10nM TMR-Star, in *P. gingivalis*, *S. gordonii* or *S. gordonii*-*P. gingivalis* biofilms.

Typical auto-correlation curves generated from intensity fluctuations of AlexaFluor555-conjugated IgG anti-rabbit antibody are presented in Fig 4(a). Curves obtained from mono-species *S. gordonii* biofilms were not modified as compared with PBS. Addition of *P. gingivalis* to *S. gordonii* biofilms did not change the shape of the curves. In contrast, for the mono-species *P. gingivalis* biofilms, auto-correlation curves were profoundly affected and distorted. Diffusion of AlexaFluor555-conjugated antibody IgG anti-rabbit antibody in PBS was estimated at 50 *μ*m^2^/sec in our experimental conditions, with a diffusion time of 0.26 msec. Results presented in Table 3 represent the ratio between diffusion coefficients calculated in biofilms as compared with the one in PBS. As already visible from the raw auto-correlation curves, ratios were modified only for monospecies *P. gingivalis* biofilms and only at low thicknesses. However, due to the great dispersion of diffusion values, ratios were not significantly decreased. Typical auto-correlation curves generated from intensity fluctuations of TMR-Star are presented in Fig 4(b) and show that all curves were distorted as compared with PBS. Diffusion of TMR-Star in PBS was estimated to be 500 *μ*m^2^/sec in our experimental conditions, with a diffusion time of 0.025 msec, which corresponds to 10 lower values than with AlexaFluor555-conjugated IgG anti-rabbit antibody. Ratios between diffusion coefficients in biofilm as compared to PBS were all below 1, except for *S. gordonii* biofilms, for which great heterogeneity was observed (Table 3). All biofilms containing *P. gingivalis* exhibited diminished diffusion coefficients, with ratios around 0.3 to 0.4, and displayed low anomality coefficients, which reflect disturbed diffusion of TMR-Star.

**Table 3:** Diffusion parameters in biofilms.

	*P. gingivalis*	*S. gordonii*	*P. gingivalis* + *S. gordonii*
	D_*biofilm*_/D_*PBS*_		
AlexaFluor555-conjugated IgG	0.74 ± 0.56	1.33 ± 0.37	1.33 ± 0.25
TMR-Star	0.23 ± 0.38	0.90 ± 1.17	0.43 ± 0.33
	Anomality coefficient (*)		
AlexaFluor555-conjugated IgG	0.79 ± 0.21	0.68 ± 0.15	0.97 ± 0.06
TMR-Star	0.42 ± 0.09	0.40 ± 0.35	0.34 ± 0.11

Diffusion parameters were calculated using SymphoTime software from auto-correlation curves established on FCS data obtained on 48 hours-biofilms of *P. gingivalis*, *S. gordonii*, or *P. gingivalis*/*S. gordonii* biofilms at 5 *μ*m deepness. Dbiofilm/DPBS corresponds to the ratio of diffusion coefficients in biofilms versus PBS. (*) The anomality coefficient is 1 for PBS.

### 2.2 Determination of biological parameters

#### 2.2.1 Experimental determination of bacterial growth parameters

Growth parameters necessary for the mathematical model were determined experimentally for the two bacteria species in BHIe medium. *c*_*s*0_ in BHIe medium were respectively of 16 *g*.*L*^−1^ for proteins for *P. gingivalis* and 2 *g*.*L*^−1^ for glucose for *S. gordonii*. The minimal substrate concentration required to allow survival of bacteria without growth is estimated to be 1.6 *g*.*L*^−1^ of proteins for *P. gingivalis* and 0.04 *g*.*L*^−1^ of glucose for *S. gordonii*.

SEM experiments performed on isolated cells allowed us to estimate diameters of bacteria before division (max) and after division (mean). From these values and with an estimation of bacterial density of *ρ* = 230 *g*.*L*^−1^, the volume and the dry weight of each species were calculated (Table 4). Using the percentage of volume occupied by bacteria in a biofilm, the volume and the dry weight of a bacterium (mean and max), the maximum biomass concentration $c_{xm}^{\left(i\right)}$ can be calculated: 38 *kg*_*x*_ *m*^−3^ for *P. gingivalis* and 44 *kg*_*x*_ *m*^−3^ for *S. gordonii*. In addition, numbers of bacteria produced within 24 hours were evaluated by PCRq. Protein consumption in bacterial culture was measured after 24h. From published data already obtained on *S. gordonii* [36], it was estimated that, in our experimental conditions, glucose was completely consumed after 24h by *S. gordonii*. These data combined to bacterial size parameters of Table 4 allowed the estimation of the yield coefficients *Y*_*xs*_ for *P. gingivalis* and *S. gordonii* (Table 5).

**Table 4:** Size parameters of *P. gingivalis* and *S. gordonii* estimated from SEM experiments.

	Length	Diameter	Volume	Dry weight
	L (*μm*)	D (*μm*)	v (*μm*^3^)	m (*g*)
*P. gingivalis*—max	1.17	0.40	0.13	3.00 ⋅ 10^−14^
*P. gingivalis*—mean	0.78	0.40	0.08	1.87 ⋅ 10^−14^
*S. gordonii*—max	0.80	0.50	0.124	2.86 ⋅ 10^−14^
*S. gordonii*—mean	0.50	0.50	0.065	1.50 ⋅ 10^−14^

$v=\frac{\pi }{4}(L-\frac{D}{3})D^{2};\;m=\rho \;v\;\text{with}\;\rho =2.3\cdot 10^{-13}g.\mu m^{-3}.$

**Table 5:** Estimation of yield and maintenance coefficients of *P. gingivalis* and *S. gordonii*.

	Number of bacteria produced by 24h	Dry weight of bateria produced by 24h (*g*_*x*_)	Substrate consumed by 24h (*g*_*s*_)	Yield coefficient *Y*_*xs*_ ($g_{x}g_{s}^{-1}$)
*P. gingivalis*	2.88 ⋅ 10^10^	5.39 ⋅ 10^−4^	3 ⋅ 10^−3^	0.18
*S. gordonii*	5.02 ⋅ 10^9^	7.53 ⋅ 10^−5^	2 ⋅ 10^−3^	0.038

For each species, the maximum specific growth rates $\mu_{m}^{\left(i\right)}$, the half saturation coefficient $K_{s}^{\left(i\right)}$ and the maintenance coefficient $m_{s}^{\left(i\right)}$ were calculated from growth curves established by measures of OD at 600 *nm* in mono species bacterial cultures at different dilutions of BHIe (Table 6). The following growth model was considered

$$\begin{array}{c} \frac{dc_{x}^{\left(i\right)}}{dt}\left(t\right)=\mu^{\left(i\right)}c_{x}^{\left(i\right)}\left(t\right),\;\;\text{with}\;\mu^{\left(i\right)}=\left(\mu_{m}^{\left(i\right)}+\theta^{\left(i\right)}\right)\frac{c_{s}^{\left(i\right)}}{K_{s}^{\left(i\right)}+c_{s}^{\left(i\right)}}-\theta^{\left(i\right)} \end{array}$$

where $\theta^{\left(i\right)}=m_{s}^{\left(i\right)}Y_{xs}^{\left(i\right)}$. An inverse problem was solved to obtain the best values for $\mu_{m}^{\left(i\right)}$, $K_{s}^{\left(i\right)}$ and *θ*^(*i*)^ which fit data of Table 6 such that

$$\begin{array}{c} \frac{1}{\mu^{\left(i\right)}}≃\frac{\frac{K_{s}^{\left(i\right)}}{c_{s}^{\left(i\right)}}+1}{\mu_{m}^{\left(i\right)}-\theta^{\left(i\right)}\frac{K_{s}^{\left(i\right)}}{c_{s}^{\left(i\right)}}}. \end{array}$$

Value of $m_{s}^{\left(i\right)}$ was deduced from values of *θ*^(*i*)^ and $Y_{xs}^{\left(i\right)}$. Results are reported in Table 1.

**Table 6:** Experimental growth parameters for *P. gingivalis* and *S. gordonii*.

BHIe dilution factor	None	2	3	4	5	10	50
$c_{s}^{\left(1\right)}$ protein (*g*.*L*^−1^)	16	8	5.33		3.2		
$c_{s}^{\left(2\right)}$ glucose (*g*.*L*^−1^)	2	1		0.5	0.4	0.2	0.04
*μ*^(1)^ *P. gingivalis* (h^−1^)	0.1575	0.1316	0.0914		0.0625		
*μ*^(2)^ *S. gordonii* (h^−1^)	0.9012	0.8735		0.7961	0.7178	0.6021	0.1448

#### 2.2.2 Estimation of damage parameters using mono-species biofilms

As damage parameters for each species (*α*^(*i*)^, *β*^(*i*)^, *γ*^(*i*)^ and *δ*^(*i*)^) could not be obtained experimentally, these values were estimated on the basis of experimental mono-species biofilms data obtained at substrate concentrations of 16 g.L^−1^ for proteins and 2 g.L^−1^ for glucose.

For a 2D model and flat biofilms, the simulated value of the biomass is fully correlated to the mean thickness. Mean thickness and roughness coefficient were therefore used to fit damage parameters. Mean thickness, measured at 3h, 24h and 48h, was used but only the value of roughness at 48h is useful because the previous values were very dependent on the unknown initialization.

**Fig 5:**
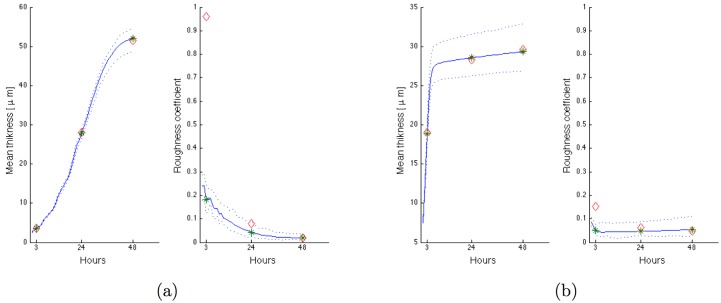
Biofilm growth simulation for mono-bacterial biofilm. Mean of 100 simulations in blue line with green star for the hours of experimental measurements, dotted lines for curves of minimum and maximum values, red diamonds for experimental data: (a) *P. gingivalis* with proteins, (b) *S. gordonii* with glucose.

The rate of damage removal *β*^(*i*)^ and the first coefficient of damage effect *γ*^(*i*)^ were arbitrarily fixed, while *α*^(*i*)^ and *δ*^(*i*)^ parameters were fitted for each species thanks to the data of the monospecies biofilms. Results are reported in Table 2. The initial number of elements containing biomass was adjusted to fit the simulated value of the mean thickness at 3 hours to the experimental value presented on Fig 3. Results of simulations with the estimated parameters are presented on Fig 5 and show a good adequacy with the experimental data.

### 2.3 Simulation of bi-bacterial biofilm growth

In order to explore the nature of interactions between *P. gingivalis* and *S. gordonii*, the growth of two-species biofilms were simulated by three different mathematical models and compared with experimental data obtained at substrate concentrations of 16 g.L^−1^ for proteins and 2 g.L^−1^ for glucose. For each hypothesis, two different criteria were used to compare the simulations results to the experimental data: the mean thickness of the biofilm and the proportion of each species in two species biofilms.

**Fig 6:**
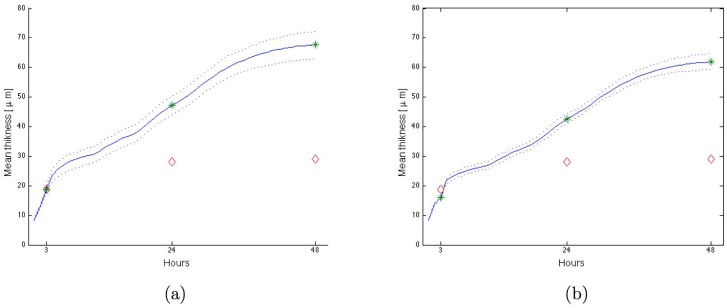
Biofilm growth simulation for bi-bacterial biofilm with independence or competition model. Mean of 100 simulations in blue line with green star for the hours of experimental measurements, dotted lines for curves of minimum and maximum values, red diamonds for experimental data, initial proportion corresponding to the initialization of the mono-bacterial biofilms (36% for *P. gingivalis*: $N_{0}^{\left(1\right)}=144$, $N_{0}^{\left(2\right)}=261$): (a) *P. gingivalis* and *S. gordonii* independent, (b) *P. gingivalis* and *S. gordonii* in competition for nutrient.

The first model, based on an independency of bacterial species for substrate, used Eqs (1)–(4). In this model, proteins and glucose were substrates of respectively *P. gingivalis* and *S. gordonii*. Two-species biofilms thicknesses were equivalent to the sum of two mono-species biofilms (see Figs 5 and 6(a) with an initial proportion of each bacterium corresponding to the initialization of the mono-bacterial biofilms). However, using this model, thickness of the simulated biofilm was higher than in experimental two-species biofilms (Fig 6(a)). Therefore, growth of the two bacteria species cannot be considered as independent for the substrate.

A frequent interaction between two species relies on competition for substrate. In the second model, also based on Eqs (1)–(4), proteins were hypothesized to be the same substrate for both *P. gingivalis* and *S. gordonii*. Assuming an identical growth yield coefficient for both species with a common substrate, the following modified parameters were obtained for *S. gordonii*:

$$\begin{array}{ccc} & Y_{xs}^{\left(2\right)}=0.18\;kg_{x}\;kg_{s}^{\left(-1\right)};\;K_{s}^{\left(2\right)}=0.8727\;kg_{s}\;m^{-3};\;m_{s}^{\left(2\right)}=1.03\cdot 10^{-4}kg_{s}\;kg_{x}^{\left(-1\right)}\;s^{-1}; & \\ & D_{s}^{\left(2\right)}=5\cdot 10^{11}\;m^{2}\;s^{-1};\;\delta^{\left(2\right)}=1.61\cdot 10^{-2}\;kg_{w}\;m^{-3} \end{array}$$

Comparison between simulated and experimental two-species biofilms is presented on Fig 6(b). With the same initialization as in the first model, thickness of simulated two-species biofilm was still higher than thickness obtained in experimental biofilms. However, the behavior is similar to the first model (independence) because of the abundance of the substrate. The interaction between the bacteria cannot therefore be considered as a competition process for the same substrate.

**Fig 7:**
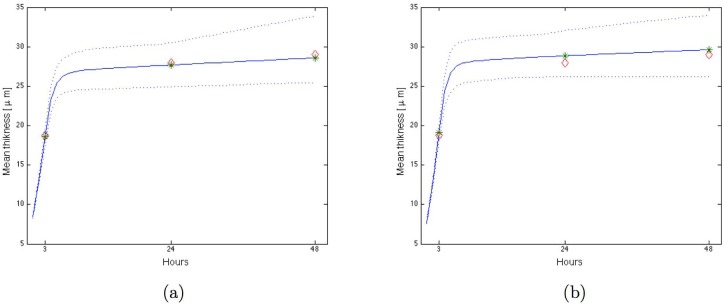
Biofilm growth simulation for bi-bacterial biofilm with toxic substance. Mean of 100 simulations in blue line with green star for the hours of experimental measurements, dotted lines for curves of minimum and maximum values, red diamonds for experimental data. (a) Initial proportion corresponding to the initialization of the mono-bacterial biofilms (36% for *P. gingivalis*: $N_{0}^{\left(1\right)}=144$, $N_{0}^{\left(2\right)}=261$) give a final proportion of 14% for *P. gingivalis* and 86% for *S. gordonii*, (b) Initial proportion of 2% for *P. gingivalis* ($N_{0}^{\left(1\right)}=7$, $N_{0}^{\left(2\right)}=315$) give a final proportion of 0.6% for *P. gingivalis* and 99.44% for *S. gordonii*.

In the third model, based on Eqs (1), (2), (4)–(6), a substance produced by *S. gordonii*, toxic for *P. gingivalis*, was introduced. The two parameters *η*^(2)^ and *ζ*^(2)^ having the same effect in the model, *ζ*^(2)^ value was set at 1 and the toxic substance production factor *η*^(2)^ was adjusted to $2.5\cdot 10^{-14}kg_{v}\;kg_{x}^{-1}\;s^{-1}$ thanks to data of experimental two-species biofilms (see Fig 7(a) where the mean thickness of the simulated biofilms fits the experimental thickness). Using this model, the final proportion of *P. gingivalis* (14%) in two-species biofilms was decreased by the action of the toxin. Yet, *P. gingivalis* amounts were still higher than those obtained in experimental biofilms. Amounts of bacteria in biofilms depend on the initial adhesion process. Therefore, if the substance produced by *S. gordonii* is toxic in initial steps of biofilm development, the initial number $N_{0}^{\left(1\right)}$ of biomass-containing elements must also be lower than in mono-species biofilms. The Fig 7(b) shows the results for an initialization with only 2% for *P. gingivalis*, which gave a final proportion of 0.6% for *P. gingivalis* in agreement with experimental data. If the same initialization was used in the first two models (independence or competition models), simulated biofilms did not fit with experimental data. The third model was therefore the best model to describe two species *P. gingivalis*-*S. gordonii* biofilms. The growth of *P. gingivalis* being limited by the accumulated damages in the bacteria, the effect of *S. gordonii* on the growth of *P. gingivalis* could be taken into account by a new value of *α*^(1)^, specific to the two-species biofilm. With *α*^(1)^ = 5.56 ⋅ 10^−5^ instead of 2.78 ⋅ 10^−5^ model 1 gave similar results than model 3. The interest of the model 3 is to test the validity of a type of interaction: the production by *S. gordonii* of a substance limiting the growth of *P. gingivalis* by the increase of the damages. It is also more accurate spatially because the concentration of limiting substance depends on proximity of *S. gordonii* bacteria.

### 2.4 Effect of substrate concentration on biofilm growth

Biofilm simulations were performed at different substrate concentrations. Experimental qPCR quantification of species were performed in 48h mono or two-species biofilms grown in non-diluted and 5-times diluted BHI.

#### 2.4.1 Mono-bacterial biofilms

**Fig 8:**
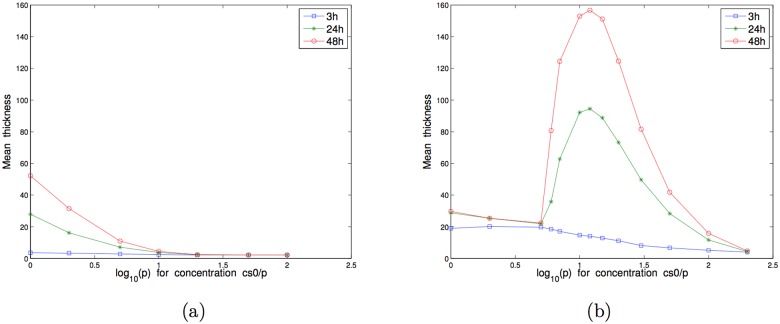
Mono-bacterial biofilm growth simulations. Mean of 50 simulations of the mean thickness at 3h, 24h and 48h with respect to the concentration of nutrients $c_{s0}^{\left(i\right)}/p$ for different values of *p*: (a) *P. gingivalis* with $N_{0}^{\left(1\right)}=175$, (b) *S. gordonii* with $N_{0}^{\left(2\right)}=315$.

As shown on Fig 8(a), when substrate concentration decreased, *P. gingivalis* biofilm thickness decreased for 3, 24 or 48 hours-biofilms. In *S. gordonii* biofilms simulations (Fig 8(b)), a similar pattern was observed for 3 hours-biofilms, with a concomitant reduction of thickness with concentration. For 24 and 48 hours-biofilms, three different parts of biofilm thicknesses curves could be observed according to substrate concentrations. At high concentrations (from pure to 5-times diluted BHI), mean thicknesses decreased slowly. When substrate concentration was decreased by a 6 to 12-times BHI dilution, mean thickness increased. Over 15-times BHI dilution, mean thicknesses were reduced. For both mono bacterial biofilms at 48h, qPCR quantification confirmed that bacteria amounts were lower in 5-times diluted BHI than in non-diluted BHI.

#### 2.4.2 Two-species biofilms

Simulations were performed with the 3 different biofilm models in two-species biofilms: independence for substrate, competition for substrate and production by *S. gordonii* of substance toxic for *P. gingivalis*.

**Fig 9:**
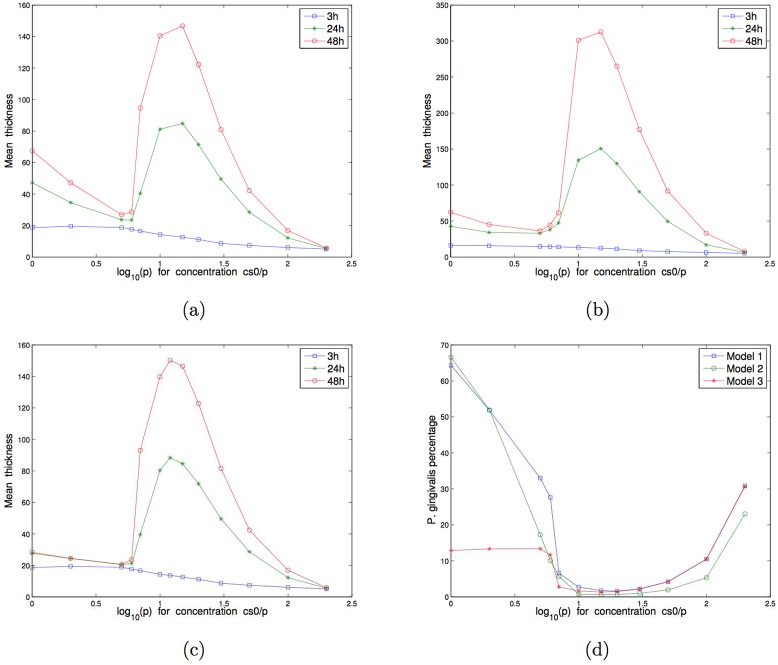
Two-species biofilm growth simulations with $N_{0}^{\left(1\right)}=144$ and $N_{0}^{\left(2\right)}=261$. Mean of 50 simulations of the mean thickness at 3h, 24h and 48h with respect to the concentration of nutrients $c_{s0}^{\left(i\right)}/p$ for different values of *p*: (a) Model 1 for *P. gingivalis* and *S. gordonii* independent, (b) Model 2 for *P. gingivalis* and *S. gordonii* in competition for proteins, (c) Model 3 for *P. gingivalis* and *S. gordonii* with production of toxic substance. (d) *P. gingivalis* percentage in biofilms for the 3 models.

For the independence model, as shown in Fig 9(a), three different parts of mean thicknesses curves were observed: in the first part (from pure to 5-times diluted BHI), mean thickness decreased with concentration. At 5-times diluted substrate concentration, quantification of species by qPCR showed that bacteria amounts decreased as compared with non-diluted BHI. Moreover *P. gingivalis* and *S. gordonii* amounts quantified by qPCR were similar to proportions obtained with independence model simulations (33% et 67%, see Fig 9(d)). In the intermediary part of the simulated curves (from 6 to 12-times diluted BHI), thickness increased, and in the last part (over 15-times diluted BHI), mean thickness decreased. In these biofilms, *P. gingivalis* was the most represented species for high substrate concentrations until 5-times dilution. Between 6 and 30-times BHI dilutions, *P. gingivalis* proportions decreased before an increase at concentrations over 50-times dilutions.

For the competition model, as shown in Fig 9(b), the shape of the curves was identical to the independence model but with higher values in the intermediary part because of the higher substrate concentration (proteins) for *S. gordonii*.

In the last biofilm model, with the production of a substance by *S. gordonii* toxic for *P. gingivalis*, the shape of mean thicknesses curves was identical to the independence model, except for the first three concentrations for which mean thickness and *P. gingivalis* proportion were low as compared with the independence model, see Fig 9(c).

**Fig 10:**
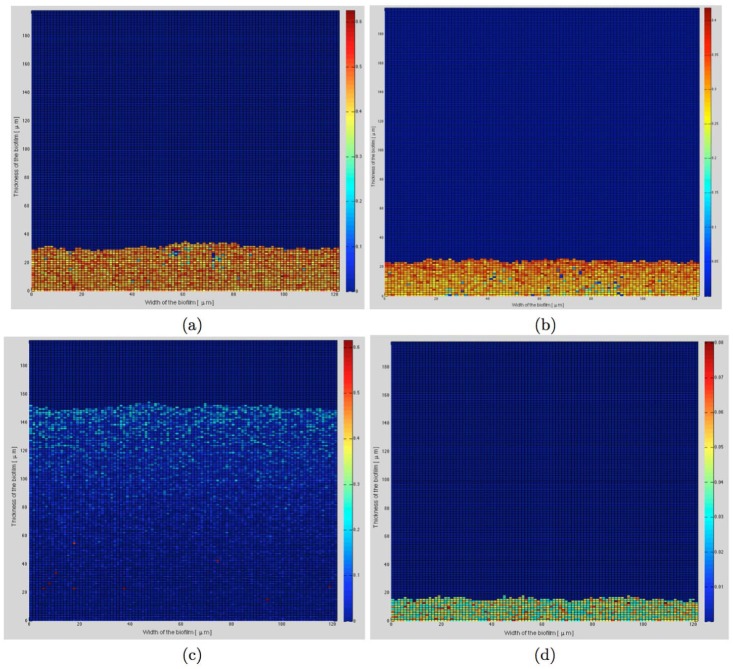
Damages in biofilm at 48h for 4 different concentrations of substrates. (a) $c_{s0}^{\left(i\right)}$, (b) $\frac{c_{s0}^{\left(i\right)}}{5}$, (c) $\frac{c_{s0}^{\left(i\right)}}{10}$, (d) $\frac{c_{s0}^{\left(i\right)}}{100}$. Damages level are depicted by colors from blue to red.

On Fig 10 are presented the distribution of damages in two-species biofilms at 48h depending on 4 different concentrations of the 2 substrates and simulated with the third model. At $c_{s0}^{\left(i\right)}$ concentration, damages are evenly distributed throughout the whole deepness of the biofilm because of the abundance of substrates in the biofilm. At the same concentration divided by 10, the biofilm is 5 times thicker and the damages are concentrated at the top of the biofilm, where substrates are more available and growth of bacteria is more important. In these conditions, there is less substrate for bacteria so that not only growth but also damages are decreased, allowing the biofilm to grow for extended times. When the concentration of substrate is divided by 100, damages are limited but the mean thickness is also almost divided by 2, due to lack of nutriments for bacteria.

## 3 Discussion

The present study was designed to develop an adequate mathematical model for two-species biofilms to study interactions between both partners in these biofilms. *S. gordonii* and *P. gingivalis* were chosen in this study because of their known interaction capacities, especially co- adherence properties. *S. gordonii*, as a primary colonizer of the oral biofilm, allows the adherence of *P. gingivalis* into the biofilm [9].

All published studies regarding heterotypic communities of *S. gordonii* with *P. gingivalis* focused on the initial adhesion step of biofilm formation. In these studies, experiments were performed in medium without nutriments (PBS) and biofilms were analyzed by confocal microscopy at times ranging from 30 minutes to 4 hours after inoculation of bacteria, under flowing conditions or not. Data obtained from these published studies allowed to understand adherence mechanism and to highlight molecules involved in co-adherence of both species [10, 21, 23]. The purpose of the present mathematical simulation was to understand the following steps of biofilm development, especially growth step. To this aim, biological and simulated experiments were performed in nutriments enriched-medium and times of analysis were chosen from 3 to 72h after inoculation of bacteria. In a first approach, biofilm formation was studied under static conditions (without flow) to reduce the number of variable parameters of the mathematical modeling.

Diffusion parameters were set up from values obtained in experimental biofilms. Experiments performed with AlexaFluor555-conjugated IgG anti-rabbit antibody and TMR-star in PBS confirmed that small molecules such as TMR-Star move faster than antibodies, as diffusion is dependent on molecule size [37].

In all three types of biofilms, diffusion coefficients of protein nature molecules such as the AlexaFluor555-conjugated antibody was not significantly modified, even if auto-correlation curves were very distorted in mono-species *P. gingivalis* biofilms. This phenomena could be explained by the capacity of *P. gingivalis* to cleave proteins thanks to specific proteases such as the gingipaïns Rgp et Kgp [38]. Degradation of proteins induced the release of fluorescent products, which mixed with native proteins and exhibited an altered profile of intensity fluctuations as measured by FCS. The coefficient diffusion for protein substrate was therefore set up at the same value as the one obtained for AlexaFluor555-conjugated IgG anti-rabbit antibody in PBS for numerical simulation.

Diffusion coefficients of TMR-Star, supposed to mimic small molecules such as peptides or sugars, was altered in both biofilms types containing *P. gingivalis*, especially in mono-species *P. gingivalis* biofilms. The decrease of diffusion could be a consequence of the production of specific biofilm extracellular matrix molecules in *P. gingivalis* biofilms, which would interfere with diffusion [39]. The nature and/or composition of matrix molecules was not studied in this work but would be of interest to better characterize the effect of bacterial species on biofilms. This extracellular matrix is indeed of great importance in the establishment of interactions between bacterial cells [40]. However, the TMR-Star diffusion was only used to estimate diffusion of sugar for *S. gordonii*. As this diffusion coefficient was not profoundly modified in *S. gordonii* containing biofilms, the coefficient diffusion for sugar substrate was therefore set up at the same value as the one obtained for TMR-Star in PBS for numerical simulation.

Simulations using mathematical model were first performed in mono-bacterial biofilms to set up biological parameters. Comparison with experimental biofilms showed that growth and substrate parameters were not sufficient to adequately reproduce mono-bacterial experimental biofilms. Indeed, differences in growth kinetics for the two kinds of mono-bacterial biofilms could be attributed to differences in intrinsic bacterial growth rate, which is faster for *S. gordonii* than *P. gingivalis*. However, to adjust thicknesses simulated data to biological experimental results, it was necessary to introduce a damage parameter in the mathematical model. Damage concentrations are supposed to increase and to accumulate with bacterial growth and to limit biofilm growth. This damage-induced effect stopped the *S. gordonii* biofilm growth before 24h whereas for *P. gingivalis* biofilms this effect appeared only near 48h. As a consequence, *P. gingivalis* biofilms were thicker than *S. gordonii* biofilms at 48h in non diluted medium. Quorum sensing, a well-established biological mechanism of communication in bacteria, could be assimilated to such kind of damage parameter as the concentration of quorum sensing molecules increase as the cellular bacteria density increased and act as a signal for bacteria to decrease growth [41]. This could explain, at least in part, the differences of behavior in mono-bacterial biofilm growth between *P. gingivalis* and *S. gordonii*.

In *P. gingivalis* mono-species biofilms simulations, substrate concentration was the main limiting factor of biofilm growth and had a direct effect on mean thickness. In contrast, in *S. gordonii* biofilms, damages induced by substrate consumption had a major impact on biofilm growth: at high concentrations, damages were important enough to inhibit biofilm growth. In the 6-12 times dilution range of substrate concentrations, damages were reduced and *S. gordonii* biofilms could grow better. Finally, in the 15-200 dilution range, substrate concentrations were limiting for *S. gordonii* growth and thickness decreased.

In a second step, two-species biofilms simulations were performed in comparison to experimental biofilms in non diluted medium. Experimental two-species biofilms were characterized by a strong predominance of *S. gordonii* over *P. gingivalis*, and the architecture of these biofilms was very close to *S. gordonii* mono-species biofilms. The first mathematical model tested was based on the hypothesis of an independent growth of each species in the biofilm, with each species consuming its own nutrient, and with a non-limiting high abundance of both nutrients in the medium. In this case the simulated bi-bacterial biofilm was equivalent to the sum of the two mono-bacterial biofilms with two times more amounts of *P. gingivalis* than *S. gordonii*. However, neither mean thickness nor proportion of *P. gingivalis* and *S. gordonii* obtained by mathematical simulation were equivalent to experimental data. Therefore, the growth of these two species biofilms cannot be considered independent.

The second mathematical model allowed to test for a hypothesis of competition of both bacteria species for the same nutrient. As nutrient concentrations are elevated in our experimental conditions, no significant difference was observed between the first and the second mathematical models. The thicknesses and proportion criteria were not fulfilled for this model. Therefore competition cannot be a model for these two species biofilm growth. This is consistent with knowledge of *P. gingivalis* and *S. gordonii* metabolism, which are dependent on proteic or carbohydrate sources respectively [15, 16].

Simulations were also performed with different substrate concentrations. Due to high substrate concentrations, mean thicknesses simulations obtained with the competition model until 5-times BHI dilutions were similar to independence model. However, differences were observed at higher BHI dilutions, mainly due to the availability of the substrate for *S. gordonii*. Curves were mainly representative of *S. gordonii* behavior.

The third model was similar to the independence model regarding mean thickness at reduced substrate concentrations but different at higher substrate concentrations. In non diluted BHI, both mean thickness and bacteria proportions are in agreement with experimental data. Differences of species proportions in this model were explained by the production of a toxic substance by *S. gordonii* for *P. gingivalis*. The fact that mean thickness was low at high substrate concentrations reflected the lower growth rates of both *P. gingivalis* and *S. gordonii* at these concentrations, explained respectively by the production of toxic substance for *P. gingivalis* and damages at these concentrations for *S. gordonii*.

The only mathematical model tested that accurately fitted with experimental data was the third model which introduced the production of a toxic substance for *P. gingivalis* produced by *S. gordonii*. The third mathematical model was therefore the best model to describe two species biofilms. Indeed, previous work on two species *P. gingivalis*/*S. gordonii* biofilms compared to mono-species *S. gordonii* biofilms demonstrated that the presence of *P. gingivalis* was able to induce a shift of the energy metabolism in *S. gordonii* [24]. By-end products of *S. gordonii* fermentation were shifted towards lactate production, which was responsible for a decreased pH. Such acidic conditions are not known to be favorable for *P. gingivalis* growth. It is also known that *S. gordonii* can produce hydrogen peroxide [42], which could be detrimental to *P. gingivalis*. Indeed, as an anaerobic bacteria, *P. gingivalis* is highly sensitive to reactive oxygen species such as hydrogen peroxide [43]. Therefore the toxic substance production could be represented by H_2_O_2_ production. *P. gingivalis* proteome was compared in the presence or absence of *S. gordonii* during the adhesion process of the biofilm development [25]. However, data were difficult to interpret because of the presence of another species, *Fusobacterium nucleatum*, besides *S. gordonii*. Simionato and coll. [44] compared the transcriptome of *P. gingivalis* in the presence of *S. gordonii* or of *S. mutans*. This study and our personal data showed that genes involved in oxidative stress response were induced in *P. gingivalis* in the presence of *S. gordonii*. It would be of great interest to measure production of hydrogen peroxyde along with expression of genes allowing its production to include these data in the model. It would be interesting to test this model in a context of cooperative, or even synergistic bacterial interactions, with modifications of parameters. For example, previous studies on *P. gingivalis* and *T. denticola* interactions showed that these species establish metabolic interactions [2] in a symbiotic way. Not only *T. denticola* consumes glycine produced by *P. gingivalis* but it also stimulated glycine production by *P. gingivalis*.

## 4 Conclusions

The new mathematical biofilm model developed in this work is especially suited to study interactions between different bacteria species in two species biofilms: either independence between species, competition for substrate and production of toxic substance by one species. The last model, with production of a toxic substance, was validated by experimental data on *P. gingivalis* and *S. gordonii* species and is now available to explore different experimental conditions. For example, damage localization associated to biofilm growth was analyzed according to substrate concentrations. New species and new nutrients can be added in this model, the only limitation being the availability of the growth parameters for each species and nutrient. In this study, experimental data were obtained in rich medium (BHI). It would be interesting to use these mathematical models in minimal medium designed to allow growth of different species. However, for longer cycles of biofilm development, it will be necessary to complete the model with processes of bacteria death or detachment. In addition, to get closer to the oral environment, a small flow can be added in the reservoir.
